# 

**Published:** 1849-04-01

**Authors:** 


### BOOKS RECEIVED.—CORRESPONDENCE.

*The American Journal of Insanity.*—We have been unavoidably compelled to postpone our elaborate notice of this interesting periodical. We will make amends in our next. The Nos. for 1847-8 have duly reached us.

*The Dublin Medical Press.*

*The Provincial Medical and Surgical Journal.*

An Anonymous Poet has transmitted to us a volume, just published by Mr. Lionel Booth, Duke-street, Portland-place, entitled, "*Varieties; by a Wanderer.*" It is not strictly within our province to notice works of a miscellaneous character, not immediately bearing upon Psychology; but we cannot allow this opportunity to pass without directing attention to this elegant little volume of beautiful poetry. We are much impressed with the high moral and philosophical character of the author's sentiments. It does infinite credit to the head and heart of the author.

*The Closing Years of Dean Swift's Life, &c., by W. R. Wilde, Esq., M.R.I.A., F.R.C.S.*—We must defer our notice of this interesting work. In our next number we purpose considering the question of Dean Swift's insanity, the point mooted in the volume before us.

We are much obliged for the copy of the *Newcastle Journal*, and regret that we cannot in this number transcribe the article it contains relative to the convict Mitchell.

We have to acknowledge the receipt of the following reports, all of which will be noticed in our next:—The Fifty-second Report of the Friends' Retreat, near York, 1848; Report of the Eastern Asylum in the City of Williamsburg, Virginia, 1847; the History of Four Cases of Eclampsia, or the "Saloom" Convulsions of Infancy, by W. Newnham, Esq., Surgeon; Twenty-sixth Report of the Bloomingdale Asylum, New York; St. Luke's Hospital, Statistical Tables for 1845-6-7; the Ethnological Journal for January, February, and March; State of the Lincoln Lunatic Asylum for 1848; Report of the Crichton Institution for the Insane, Dumfries, 1848.

*On Femoral Rupture; its Anatomy, Pathology, and Surgery, by John Gay, Esq., F.R.C.S.*—An able work by an accomplished surgeon.

*On Eruptions of the Face, Head, and Hands, by T. H. Burgess, M.D.*—A valuable work, ably written, and full of sound practical advice on the treatment of skin diseases.

*On Infantile Laryngismus, by J. Reid, M.D.*, 1849.—This is the production of an experienced and accomplished physician. His views of the Pathology and Treatment of Infantile Laryngismus are entitled to the serious consideration of the profession. We can strongly recommend the work to our readers.

*M.D.*—Our remarks on the Medical Treatment of Insanity will be based on the Lectures of Sir R. Morison, M.D., edited by his son.

### APPOINTMENTS.

*Mr. Gaskell*, late Superintendent of the Lancaster Asylum, has been appointed one of the Medical Commissioners in Lunacy.

*Dr. Thurnam*, of the Retreat, near York, has been appointed Medical Superintendent of the Wiltshire Asylum, in the course of being erected.

Both these appointments are unexceptionable.

